# The association between obstructive sleep apnea and metabolic syndrome: a systematic review and meta-analysis

**DOI:** 10.1186/s12890-015-0102-3

**Published:** 2015-09-21

**Authors:** Shaoyong Xu, Yi Wan, Ming Xu, Jie Ming, Ying Xing, Fei An, Qiuhe Ji

**Affiliations:** Department of Endocrinology, Xijing Hospital, Fourth Military Medical University, 169 Changle Road West, Xi’an, 710032 China; Department of Health Statistics, School of Preventive Medicine, Fourth Military Medical University, 169 Changle Road West, Xi’an, 710032 China; Department of Cardre Ward, Lanzhou General Hospital of Lanzhou Military Region, People’s Liberation Army, 333 Binhe Road South, Lanzhou, 730050 China; Department of Otolaryngology Head and Neck Surgery, Lanzhou General Hospital of Lanzhou Military Region, People’s Liberation Army, 333 Binhe Road South, Lanzhou, 730050 China

**Keywords:** Obstructive sleep apnea, Metabolic syndrome, Meta-analysis, Cross-sectional study, Case–control study, Cohort

## Abstract

**Background:**

Obstructive sleep apnea (OSA) is characterized by repeated episodes of obstruction of the upper airway. Numerous studies have indicated a relationship between OSA and metabolic syndrome (MS), but the results remain debatable. We aimed to perform a systematic review and meta-analysis to evaluate the association between OSA and MS.

**Methods:**

We searched electronic databases (PubMed, EMBASE, and ISO Web of Knowledge) up to September 2014 with English-language restriction. Cross-sectional, case–control, and cohort studies in which the presence of OSA was assessed by objective measurements, the exposure of interest was OSA, and the outcome of interest was the presence (or incidence) of MS were included. The adjusted odds ratios (ORs) (or relative risk) and 95 % confidence intervals (CIs) were extracted and pooled. Sensitivity analyses were conducted, and heterogeneity and publication bias were assessed.

**Results:**

Overall, 15 cross-sectional (2456 patients with OSA and 1705 subjects without OSA), five case–control (1156 OSA patients and 404 controls), and no cohort studies were included. The pooled ORs of MS in individuals with OSA for cross-sectional and case–control studies were 2.87 (95 % CI: 2.41–3.42) and 2.56 (95 % CI: 1.98–3.31), respectively. There was clinically unimportant (*I*^*2*^ = 20 %) and moderate (*I*^*2*^ = 35 %) between-study heterogeneity of the analysis. The pooled crude ORs of MS in individuals with mild and moderate-to-severe OSA was 2.39 (95 % CI: 1.65–3.46) and 3.45 (95 % CI: 2.33–5.12), respectively, and there was substantial heterogeneity in the meta-analyses (*I*^*2*^ = 53 % and *I*^*2*^ = 63 %, respectively). However, no evidence of publication bias was detected.

**Conclusions:**

OSA is shown to be associated with MS, although causality between these two factors has not been demonstrated yet. Future cohort and randomized controlled studies are needed.

**Electronic supplementary material:**

The online version of this article (doi:10.1186/s12890-015-0102-3) contains supplementary material, which is available to authorized users.

## Background

Obstructive sleep apnea (OSA) is a condition in which there is repetitive obstruction of the upper airway during sleep, resulting in hypopnea (reduced airflow during sleep) or apnea (complete cessation of airflow during sleep) [1, 2]. Patients with OSA may experience symptoms, including loud souring, frequent arousals, sleep fragmentation, and daytime sleepiness, which characterize obstructive sleep apnea syndrome [2, 3]. Studies have shown that OSA is associated with increased risks of hypertension, stroke, type 2 diabetes mellitus, and other cardiovascular diseases [4–7]. Approximately 4 % of men and 2 % of women in the general population were considered as having OSA in 1993 [8, 9]. With the increasing epidemic of obesity, the prevalence of OSA among adults is estimated to be up to 17 % [10].

Metabolic syndrome (MS) is a cluster of metabolic risk factors for diabetes and cardiovascular diseases, including central obesity, hypertension, hyperglycemia, and dyslipidemia [11–13]. The prevalence of MS varies between 20 and 40 % worldwide and tends to increase in parallel with the epidemic of obesity [14, 15]. In the past decade, numerous studies have indicated a relationship between OSA and MS, but the results remain debatable [16]. Coughlin et al. found that OSA was independently associated with an increased risk of MS, irrespective of age and body mass index (BMI) [17]. However, Papanas et al. showed that the strong association between the presence of OSA and MS became non-significant when BMI was considered [18].

Based on these findings, and the fact that no meta-analysis has been conducted on this relationship, we aimed to perform a systematic review and meta-analysis to evaluate the association between OSA and MS.

## Methods

### Search strategy

We followed the guidelines of the Meta-analysis of Observational Studies in Epidemiology group in reporting the meta-analysis [19]. We searched electronic databases (PubMed, EMBASE, and ISO Web of Knowledge) up to September 2014 using the following terms: “obstructive sleep apnea (apnoea)” or “sleep apnea (apnoea) syndrome” or “sleep-disordered breathing” or “OSA” or “SAS” or “SDB” and “metabolic syndrome” or “insulin resistance syndrome” or “metabolic syndrome X” or “syndrome X” (Additional file 1). We also checked the reference lists from the included studies and relevant review articles for potential publications that might be suitable for inclusion. English-language restriction was applied.

Two reviewers (S.X. and J.M.) independently checked titles and abstracts against the eligibility criteria and obtained full-text versions of potentially relevant articles. Disagreements were discussed with a third party (Y.W. and Q.J.) before a final decision on inclusion.

### Identification of studies

Cross-sectional, case–control, and cohort studies in which the presence of OSA was assessed by standard objective measurements were included. Polysomnography (PSG), which must be performed in a sleep laboratory setting, is considered the reference standard for diagnosing OSA [20, 21]. Therefore, studies that used type IV monitors, which cannot differentiate between obstructive and central apneas, devices that cannot estimate the apnea–hypopnea index (AHI), questionnaires, or self-reported snoring to assess OSA were excluded. Studies in which the exposure of interest was OSA and the outcome of interest was the presence (for case–control and cross-sectional studies) or incidence (for cohort studies) of MS were included. For studies to be eligible for inclusion, they must have reported (or provided sufficient data to enable the calculation of) a risk estimate for MS related to OSA, together with a 95 % confidence interval (CI), a *P* value, or a standard error (SE). Literature reviews, letters, and comments were excluded. Conference reports that were not subsequently published were excluded in the main body, but included as sensitivity analyses, which are available online as Additional files.

### Data collection

Two reviewers (S.X. and J.M.) independently extracted data from all eligible studies by using a standardized extraction form (agreement was 98.5 %). The third party (Y.W. and Q.J) checked the data and resolved the discrepancies by discussing and cross-checking against the primary papers.

The data included the following: first author’s name, publication year, study type, location of study, enrollment criteria of patients, method and criteria of defining OSA and MS, sample size, mean age of the patients, percentage of male sex, mean BMI, history of smoking and drinking, number and percentage of patients in both groups, crude and adjusted odds ratios (ORs) (for case–control and cross-sectional studies) or relative risks (RRs) (for cohort studies), and adjusted confounders (if provided). The data were recorded in a preformatted Excel spreadsheet.

### Assessment of methodological quality

The methodological quality of the included studies was evaluated based on the Newcastle–Ottawa Scale (NOS) [22], by appraising the following characteristics (an example for case–control and cross-sectional studies):

1. Selection (4 items): adequacy of case definition; representativeness of the cases; selection of controls; and definition of controls.
2. Comparability (1 item): comparability of cases and controls on the basis of the design or analysis.
3. Exposure (3 items): ascertainment of exposure; same method of ascertainment for cases and controls; and non-response rate (same rate for both groups).

A star rating system was used to indicate the quality of a study, with a maximum of nine stars. A study could be awarded a maximum of one star for each numbered item within the selection and exposure categories. A maximum of two stars could be allocated for comparability; one star was allocated if the most important confounder had been adjusted for in the analysis and a second star was allocated if any other adjustments were made.

### Severity of OSA

The AHI was defined as the mean number of episodes of apnea and hypopnea per hour of sleep. OSA severity categories were defined according to commonly used clinical cutoffs as follows [21]: no OSA (AHI < 5 events/h); mild OSA (AHI ≥ 5 events/h but < 15 events/h); moderate OSA (AHI ≥ 15 events/h but < 30 events/h); and severe OSA (AHI ≥ 30 events/h). For studies that used an AHI ≥ 10 or 15 events/h as diagnosis of OSA, the severity of OSA was based on the authors’ opinion.

### Statistical analysis

All of the statistical analyses were conducted by using RevMan 5.1 software (The Nordic Cochrane Centre, The Cochrane Collaboration, 2011) or Stata 10.0 (StataCorp, College Station, TX, USA). The association between OSA and MS was assessed based on cross-sectional, case–control, and cohort studies, separately. The pooled ORs for cross-sectional and case–control studies, and RRs for cohort studies, were generated separately. The adjusted ORs (or RRs) (obesity was considered the most important factor) were preferred for the meta-analysis, and calculation of crude ORs based on the raw data was also adopted in case of the absence of adjusted ORs (or RRs). Subgroup meta-analyses were performed because of the multiple criteria of OSA (i.e., AHI ≥ 5 events/h, ≥ 10 events/h, or ≥ 15 events/h). Where results were separately reported for men and women or for mild and moderate-to-severe OSA, they were initially pooled within the study, and then a single estimate was included in the meta-analysis. In addition, a random effects model (if substantial heterogeneity was present) or a fixed effects model (if substantial heterogeneity was not present) was used to assess study heterogeneity by using the Cochrane Q-test, the *I*^*2*^ test, and the Galbraith plot (if necessary) [23]. Heterogeneity was considered to be significant at *P* < 0.10 for the Q statistic. We defined *I*^*2*^ values below 30 % as unimportant, 30–50 % as moderate heterogeneity, 51–75 % as substantial heterogeneity, and >75 % as considerable heterogeneity. Meta-regression analyses were performed to evaluate the effect of mean BMI, mean patient’s age, percentage of male sex, adjusted status (yes/no), and publication year.

Subgroup analyses were conducted to test the robustness of the findings (e.g., based on the exclusion of studies that provided unadjusted data only, or on the exclusion of studies that used different parameters in the selection of participants or MS definitions). Furthermore, the Begg’s test [24] and Egger’s test [25] were used to evaluate publication bias, which was further assessed by using funnel plots.

## Results

**Fig. 1:**
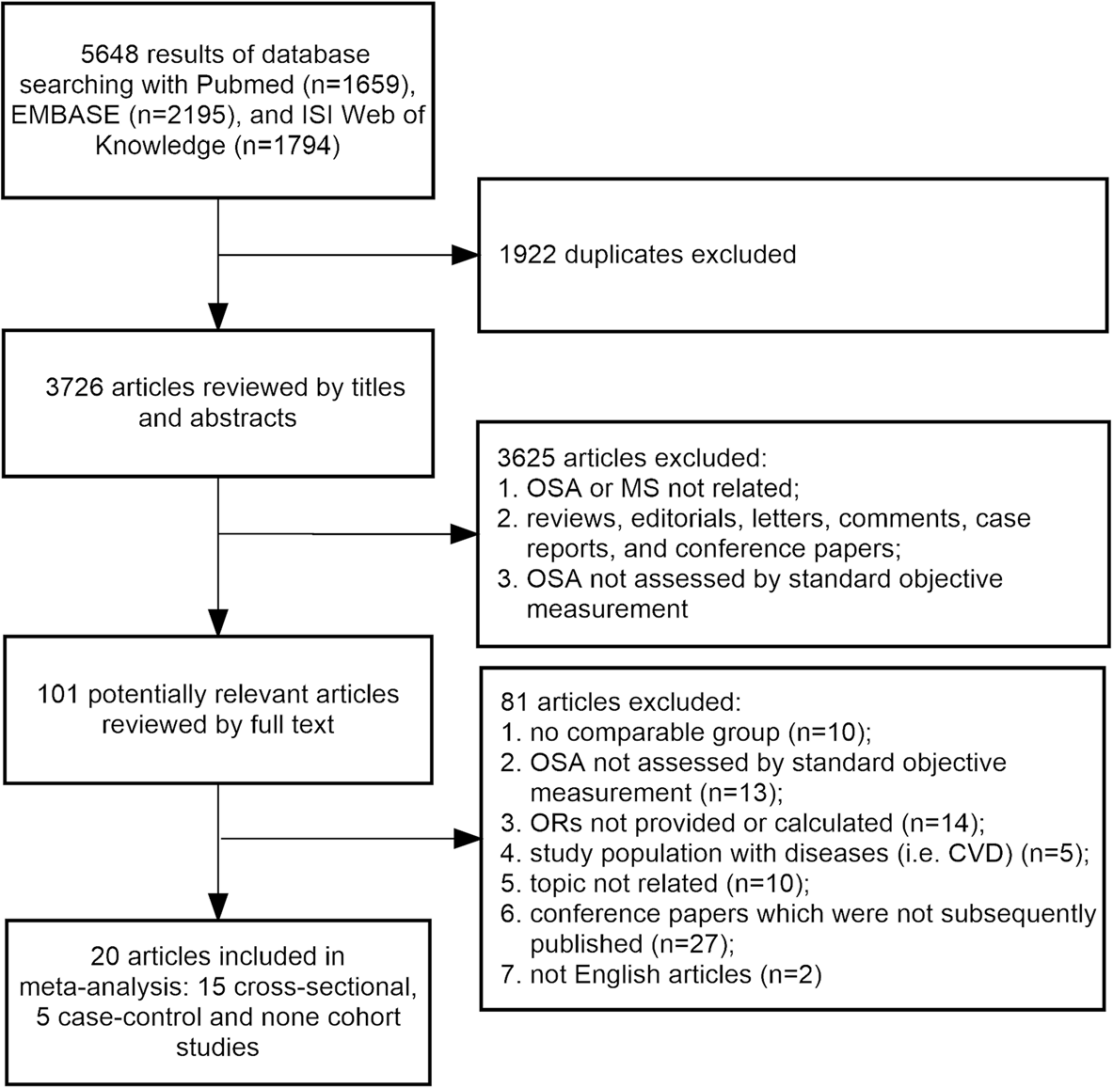
Flow chart of the process of article selection for meta-analysis. OSA: obstructive sleep apnea; MS: metabolic syndrome; CVD: cardiovascular disease

Overall, 5648 references were identified and 20 studies were finally included in the review [17, 18, 26–43]. All of the included studies were reviewed by full text. Of the included studies, 15 were cross-sectional [18, 26–33, 35–37, 40, 42, 43], five were case–control [17, 34, 38, 39, 41], and none were cohort studies. In total, 2456 patients with OSA and 1705 subjects with no OSA in cross-sectional studies, together with 1156 OSA patients and 404 controls in case–control studies, were included in the meta-analysis for the association of OSA with MS (Fig. 1).

Fourteen studies were hospital-based studies [17, 18, 28–34, 37–41], five were community-based studies [26, 35, 36, 42, 43], and one was a population-based study [27]. All of the studies used the AHI based on an overnight PSG to diagnose OSA, whereas the AHI thresholds differed between studies. Two studies [44, 45], of which one used the minimal patient contact sleep diagnosis system and another used a pulse-oximeter to assess OSA, were excluded. Almost all of the studies adopted the National Cholesterol Education Program Adult Treatment Panel III criteria to define MS, except for one study [39] in which the International Diabetes Federation criteria were used (Table 1).

**Table 1:** Characteristics of the included studies on obstructive sleep apnea and the risk of metabolic syndrome

First author, year	Study design, location	OSA diagnosis (Methods; criteria)	Definition of MS	OSA group		Non-OSA group		ORs			NOS scores
				N	% MS	N	% MS	Adjusted	OR (95 % CI)	Confounder	
Cross-sectional study											
Jamie C.M. Lam, 2006 [35]	Community-based study, Hong Kong, China	PSG; AHI ≥5 events/h.	ATP III	95	58 %	160	21 %	Yes	2.65 (1.38–5.08)	Age, gender, BMI, smoking and alcohol consumption	9
James M. Parish, 2007 [28]	Retrospective hospital-based study, United states	PSG; AHI ≥ 5 events/h or ≥10 events/h	ATP III	174	56 %	54	43 %	No	1.74 (0.94–3.22)	None	5
Susan Redline, 2007 [43]	Two-stage community-based study, United States	PSG; AHI ≥5 events/h.	Adapted ATP III	22	59 %	248	16 %	Yes	6.49 (2.52–16.70)	Age, sex, race and preterm status.	9
F. Javier Nieto, 2009 [26]	Community-based study, United states	PSG; AHI ≥5 events/h.	ATP III	253	-----	293	-----	Yes	2.37 (1.60–3.50)	Age, sex, BMI	8
Francesco Angelico, 2010 [31]	Hospital-based study, Italy	PSG; AHI >5 events/h.	ATP III	178	53.9 %	48	42.6 %	No	1.64 (0.86–3.12)	None	6
Stephen Guill, 2010 [42]	Community and web-based study, United States	PSG. AHI >5 events/h.	ATP III	12	33 %	18	28 %	No	1.30 (0.27–6.33)	None	6
Nikolaos Papanas, 2010 [18]	Hospital-based study, Greece	PSG; AHI >5 events/h	ATP III	53	71.7 %	30	36.7 %	No	4.38 (1.69–11.35)	None	6
Swastik Agrawal, 2011 [30]	Hospital-based study, Northern India	PSG; AHI >5 events/h.	ATP III	187	79 %	40	48 %	No	4.19 (2.05–8.56)	None	6
H.-W.M. Breuer, 2011 [40]	Hospital-based study, Germany.	PSG; AHI ≥ 5 events/h.	ATP III or IDF	360	47 %	58	26 %	No	2.54 (1.36–4.73)	None	6
M. Gasa, 2011 [32]	Multi hospital-based study, Spain	PSG; AHI ≥ 15 events/h.	ATP III	114	70 %	44	36 %	Yes	2.84 (1.30–6.22)	Age, gender and BMI	8
Duygu Ozol, 2011 [29]	Hospital-based study, Ankara, Turkey	PSG; AHI ≥5 events/h.	ATP III	195	23.8 %	20	10.0 %	No	2.81 (0.61–12.97)	None	6
Jenny Theorell-Haglöw, 2011 [27]	Two-stage population-based study, Sweden	PSG; AHI ≥5 events/h.	ATP III	135	44.4 %	265	16.2 %	No	4.13 (2.58–6.62)	None	7
Assoumou HG, 2012 [36]	Community-based study, France	PSG; AHI >15 events/h	ATP III	449	12.5 %	357	5 %	No	2.68 (1.55–4.65)	None	7
Qi-Chang Lin, 2012 [33]	Hospital-based study, China	PSG; AHI ≥5 events/h.	Modified ATP III	113	38.9 %	45	8.9 %	No	6.54 (2.19–19.52)	None	6
Bienvenido Barreiro, 2013 [37]	Hospital-based study, Barcelona, Spain	PSG; AHI ≥5 events/h.	ATP III	116	68 %	25	32 %	No	4.36 (1.73–11.01)	None	5
Case–control study											
Steven R. Coughlin, 2004 [17]	Hospital-based study, United kingdom	PSG; AHI ≥15 events/h	ATP III	61	87 %	43	35 %	Yes	9.1 (2.6–31.2)	Age, BMI, smoking and alcohol consumption	7
Ryujiro Sasanabe, 2006 [39]	Hospital-based study, Japan	PSG; AHI ≥5 events/h	IDF	819	47.3 %	89	16.9 %	Yes	2.10 (1.46–3.02)	Age and BMI	8
Bharat Bhushan, 2010 [34]	Hospital-based study, Northern India	PSG; AHI > 10 events/h.	ATP III	121	67.8 %	119	42.0 %	Yes	3.40 (1.93–6.05)	Age, BMI, smoking, and alcohol consumption	8
Ozen K. Basoglu, 2011 [38]	Hospital-based study, Izmir, Turkey	PSG; AHI ≥5 events/h	ATP III	36	47.2 %	34	29.4 %	No	2.15 (0.80–5.76)	None	6
A. Barcelo, 2011 [41]	Hospital-based study, Spain	PSG; AHI ≥10 events/h.	ATP III	119	38.0 %	119	21.0 %	No	2.54 (1.41–4.56)	None	5

*OSA* obstructive sleep apnea, *MS* metabolic syndrome, *OR* odds ratio, *CI* confidence interval, *NOS* Newcastle-Ottawa Scale, *AHI* apnea–hypopnea index, *BMI* body mass index, *ATP* Adult Treatment Panel, *IDF* International Diabetes Federation, *PSG* polysomnography, *M/S* moderate/severe

The NOS [22] was used to evaluate the methodological quality of the included studies (Table 2). The mean NOS score for the cross-sectional and case–control studies was 6.7 and 6.8, respectively, indicating that the overall methodological quality was generally good. Fourteen hospital-based studies had relatively low scores in the selection category, mainly because the controls who were derived from a hospitalized population (not the same community) may have resulted in potential selection bias. In the comparability category, obesity was the most important factor, but was adjusted for in only seven studies [17, 26, 32, 34, 35, 39, 43]. In the exposure category, all of the studies had perfect scores for the ideal and same method of ascertainment for cases and controls. Venous blood was obtained to assess MS in the fasting state in the morning after performing PSG in almost all of the prospective studies. Therefore, we were cautious that they had nearly a 100 % response rate for both groups. Two studies did not receive stars in the item of non-response rate [26, 28]. One of these studies was a retrospective study in which the authors mentioned that 22 patients were excluded for insufficient data, but they did not report the non-response rate in each group separately [28]. The other study was a community-based study in which a subset of participants from the Wisconsin Sleep Cohort Study were invited to measure metabolic parameters; therefore, the non-response rate for each group should have been described [26].

**Table 2:** Scores of Newcastle-Ottawa quality assessment scale

Studies	Selection				Comparability		Exposure			Total scale
	1	2	3	4	5A	5B	6	7	8	
Cross-sectional study										
Jamie C.M. Lam, 2006 [35]	Yes	Yes	Yes	Yes	Yes	Yes	Yes	Yes	Yes	9
James M. Parish, 2007 [28]	Yes	Yes	No	Yes	No	No	Yes	Yes	No	5
Susan Redline, 2007 [43]	Yes	Yes	Yes	Yes	Yes	Yes	Yes	Yes	Yes	9
F. Javier Nieto, 2009 [26]	Yes	Yes	Yes	Yes	Yes	Yes	Yes	Yes	No	8
Francesco Angelico, 2010 [31]	Yes	Yes	No	Yes	No	No	Yes	Yes	Yes	6
Stephen Guill, 2010 [42]	Yes	No	Yes	Yes	No	No	Yes	Yes	Yes	6
Nikolaos Papanas, 2010 [18]	Yes	Yes	No	Yes	No	No	Yes	Yes	Yes	6
Swastik Agrawal, 2011 [30]	Yes	Yes	No	Yes	No	No	Yes	Yes	Yes	6
H.-W.M. Breuer, 2011 [40]	Yes	Yes	No	Yes	No	No	Yes	Yes	Yes	6
M. Gasa, 2011 [32]	Yes	Yes	No	Yes	Yes	Yes	Yes	Yes	Yes	8
Duygu Ozol, 2011 [29]	Yes	Yes	No	Yes	No	No	Yes	Yes	Yes	6
Jenny Theorell-Haglöw, 2011 [27]	Yes	Yes	Yes	Yes	No	No	Yes	Yes	Yes	7
Assoumou HG, 2012 [36]	Yes	Yes	Yes	Yes	No	No	Yes	Yes	Yes	7
Qi-Chang Lin, 2012 [33]	Yes	Yes	No	Yes	No	No	Yes	Yes	Yes	6
Bienvenido Barreiro, 2013 [37]	Yes	No	No	Yes	No	No	Yes	Yes	Yes	5
Case–control study										
Steven R. Coughlin, 2004 [17]	Yes	No	No	Yes	Yes	Yes	Yes	Yes	Yes	7
Ryujiro Sasanabe, 2006 [39]	Yes	Yes	No	Yes	Yes	Yes	Yes	Yes	Yes	8
Bharat Bhushan, 2010 [34]	Yes	No	No	Yes	Yes	Yes	Yes	Yes	Yes	8
Ozen K. Basoglu, 2011 [38]	Yes	Yes	No	Yes	No	No	Yes	Yes	Yes	6
A. Barcelo, 2011 [41]	Yes	No	No	Yes	No	No	Yes	Yes	Yes	5

A star rating system was used to indicate the quality of a study, with a maximum of nine stars. A study could be awarded a maximum of one star for each numbered item within the selection and exposure categories. A maximum of two stars could be allocated for comparability

**Fig. 2:**
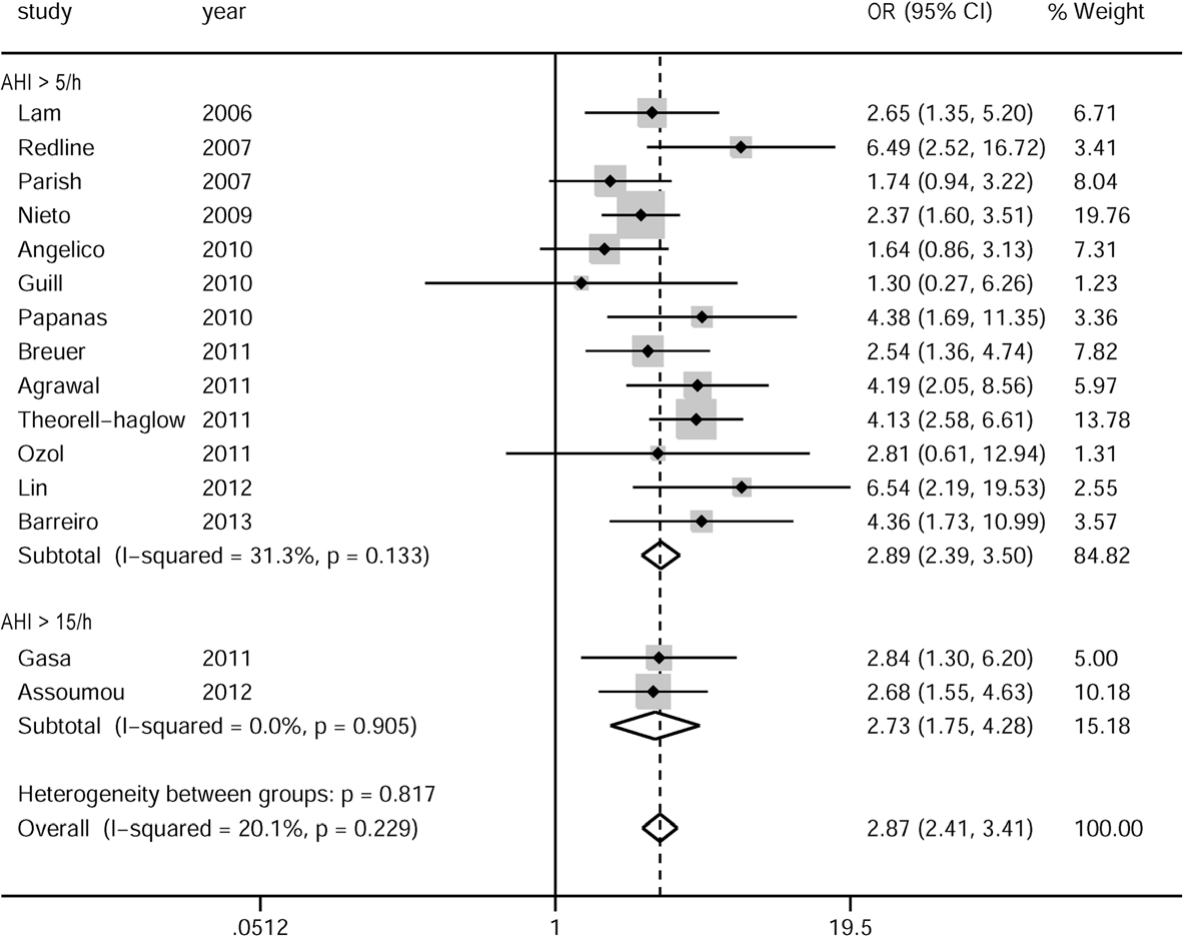
Meta-analysis of obstructive sleep apnea syndrome and the risk of metabolic syndrome for cross-sectional studies. AHI: apnea–hypopnea index; OR: odds ratio; CI: confidence interval

**Fig. 3:**
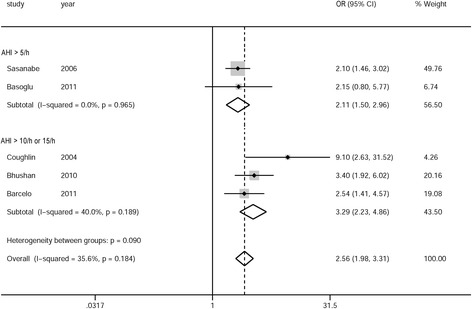
Meta-analysis of obstructive sleep apnea and the risk of metabolic syndrome for case–control studies. AHI: apnea–hypopnea index; OR: odds ratio; CI: confidence interval

As mentioned above, the adjusted ORs were preferred for the meta-analysis. For the remaining studies without adjusted ORs, the crude or calculated ORs were adopted for the meta-analysis. Sensitivity analysis was then performed based on the exclusion of studies that provided unadjusted data only. Two studies separately reported the adjusted ORs for men and women [39] or for mild and moderate-to-severe OSA [26]. The results were initially pooled within the study and then a single estimate was included in the meta-analysis. Overall, for cross-sectional studies, the pooled OR of MS in individuals with OSA was 2.87 (95 % CI: 2.41–3.42), with unimportant between-study heterogeneity of the analysis (*P* = 0.23, *I*^*2*^ = 20 %). Subgroup meta-analyses based on the criteria of OSA (AHI > 5 events/h or > 15 events/h) showed similar pooled ORs (2.89 [95 % CI: 2.39–3.50] and 2.73 [95 % CI: 1.74–4.28], respectively). There was no evidence of substantial heterogeneity in the subgroup analyses (*P* = 0.13, *I*^*2*^ = 31 % and *P* = 0.91, *I*^*2*^ = 0 %, respectively) (Fig. 2). For case–control studies, the pooled OR of MS in individuals with OSA was 2.56 (95 % CI: 1.98–3.31), with moderate between-study heterogeneity of the analysis (*P* = 0.19, *I*^*2*^ = 35 %). Subgroup meta-analyses based on the criteria of OSA (AHI > 5 events/h or > 10 events/h [15 events/h]) showed similar pooled ORs (2.11 [95 % CI: 1.50–2.96] and 3.29 [95 % CI: 2.23–4.85], respectively). The *I*^*2*^ was 0 % (*P* = 0.96) and 39 % (*P* = 0.19), respectively (Fig. 3). In addition, mixed effect, multilevel meta-analyses showed no effects of mean BMI, mean patient’s age, percentage of male sex, adjusted (yes/no), and publication year on the overall results (*P* > 0.05 for all analyses).

**Fig. 4:**
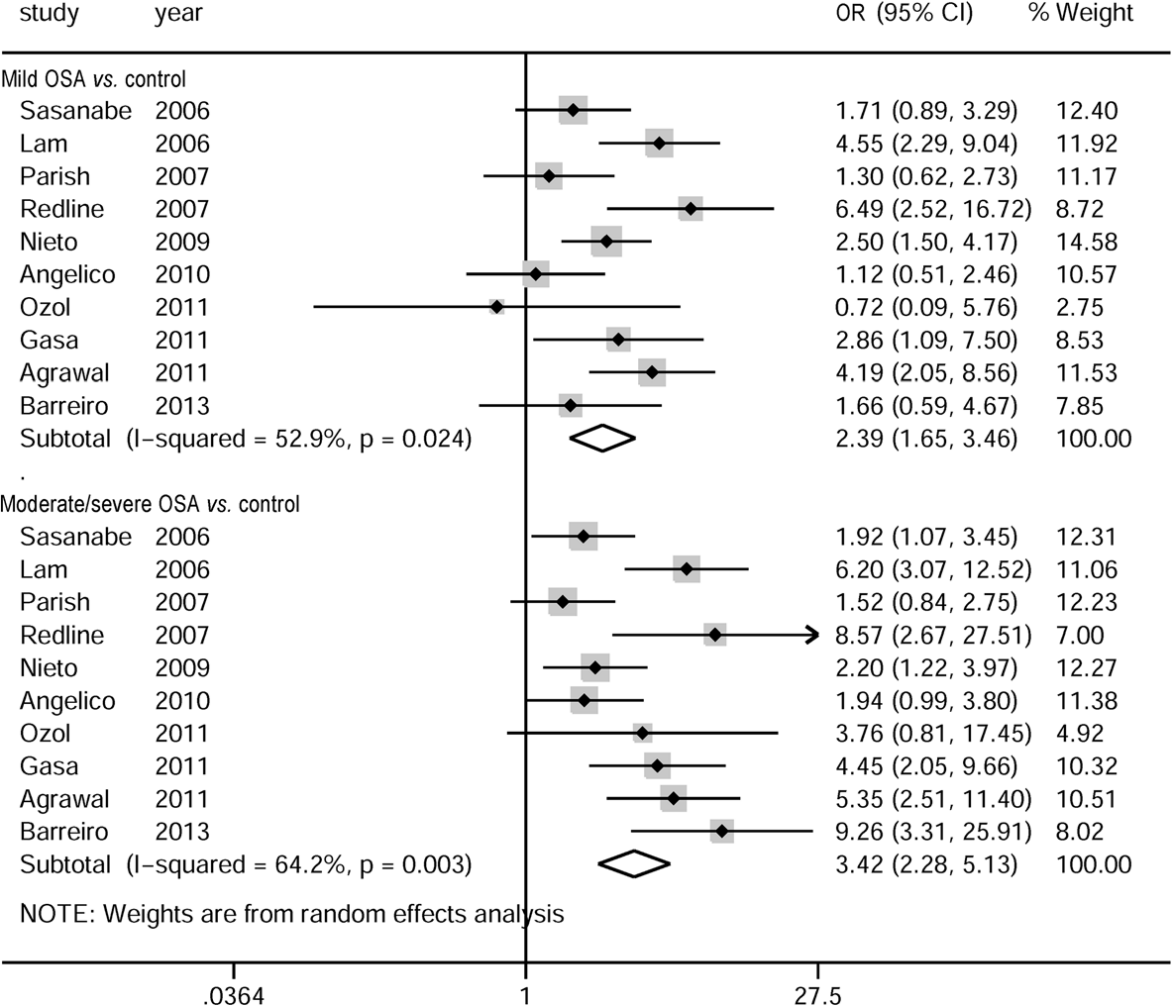
Pooled crude odds ratios of metabolic syndrome in patients with mild or moderate-to-severe obstructive sleep apnea. OSA: obstructive sleep apnea; OR: odds ratio; CI: confidence interval

Only some of the cross-sectional studies and one case–control study provided data on severity of OSA related to MS. Therefore, meta-analysis on the relationship between MS and the severity of OSA was conducted in all of the eligible studies together. The pooled crude ORs of MS in individuals with mild and moderate-to-severe OSA were 2.39 (95 % CI: 1.65–3.46) and 3.45 (95 % CI: 2.33–5.12), respectively. There was substantial heterogeneity in the meta-analyses (*P* = 0.02, *I*^*2*^ = 53 % and *P* = 0.004, *I*^*2*^ = 63 %, respectively) (Fig. 4).

To verify the robustness of the results, as well as the potential sources of heterogeneity, subgroup analyses were performed. We examined exclusion of studies with obesity as the most important unadjusted factor (Model 1), those where the Adult Treatment Panel III criteria were not used to define MS (Model 2), and those with a special population (children, young men, morbidly obese individuals, and non-obese people) (Model 3). These subgroup analyses only resulted in a marginal change in the pooled ORs (Table 3).

**Table 3:** Subgroup analyses

	Excluded studies	N	Reasons	Subgroup analysis		
				OR	*I* ^*2*^ (%)	*P* value
Model 1	Parish, Angelico, Guill, Papanas, Agrawal, Breuer, Ozol, Theorell-Haglöw, Assoumou, Lin, Barreiro, Basoglu, Barcelo	13	Obesity as the most important factor unadjusted	a: 2.74	20	0.29
				b: 2.60	67	0.05
Model 2	Sasanabe	1	IDF criteria used	a: 2.87	20	0.23
				b: 3.11	23	0.27
Model 3	Redline, Guill, Gasa, Lin	4	Special population (children, young male, morbidly obese individuals, and non-obese people)	a: 2.74	11	0.34
				b: 2.63	19	0.19

a: cross-sectional studies; b: case–control studies

*OR* odd ratio, *IDF* International Diabetes Federation

**Fig. 5:**
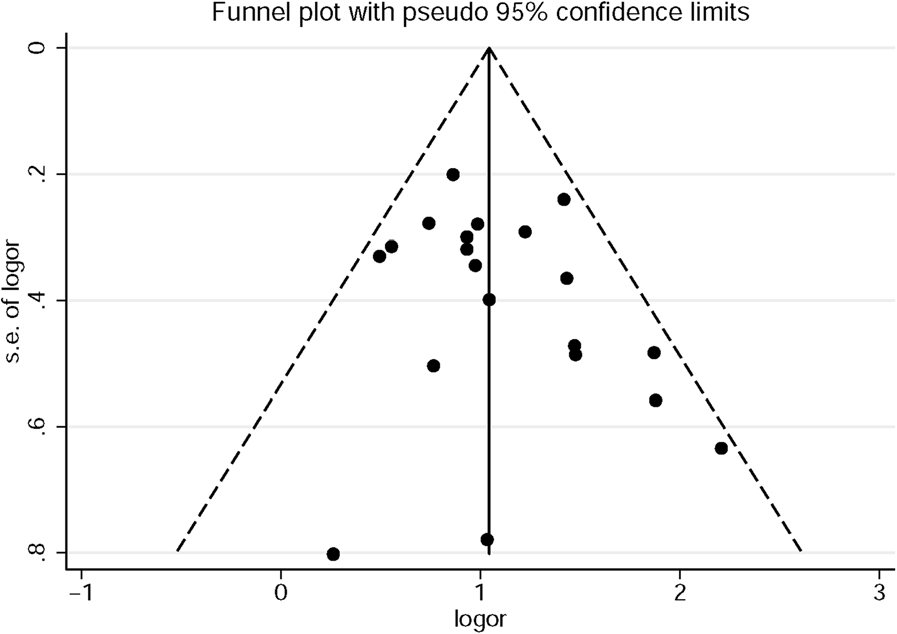
Funnel plots for assessment of publication bias among all included studies in the meta-analysis

No evidence of publication bias was detected by using Begg’s test (z = 1.18, *P* = 0.232) and Egger’s test (z = 1.18, *P* = 0.232). Visual inspection of the funnel plots showed that they were symmetrical (Fig. 5).

We also performed meta-analyses that included two conference reports that were not subsequently published [46, 47]. The results were similar to those that did not include conference reports, although there was substantial heterogeneity in the meta-analyses. The Galbraith plot showed that the conference reports greatly contributed to between-group heterogeneity (Additional file 2: Figure S1, Additional file 3: Figure S2, and Additional file 4: Figure S3)

## Discussion

Our meta-analysis, which included 15 cross-sectional (2456 patients with OSA and 1705 subjects with no OSA) and five case–control studies (1156 OSA patients and 404 controls), identified a significant relationship between OSA and MS. This meta-analysis also showed that the pooled ORs of MS in individuals with OSA for the cross-sectional and case–control studies were 2.87 (95 % CI: 2.41–3.42) and 2.56 (95 % CI: 1.98–3.31), respectively. Although there were substantial differences among studies (e.g., the diagnosis of OSA and definition of MS), subgroup analyses confirmed the robust relationship.

Obesity is strongly linked to MS and is also a well-known risk factor for OSA [48]. With the escalating prevalence of obesity, the association between OSA and MS has been increasingly recognized over the past few years [16]. Several possible mechanisms have been suggested to explain the biological plausibility of OSA, independent of obesity, increasing the risk of MS [16]. These mechanisms may be related to intermittent hypoxia (IH), oxidative stress, cytokines, and selective activation of systematic inflammation [49, 50]. Recurrent obstructive events result in IH in OSA. Repaid reoxygenation of transiently ischemic tissues can damage tissues and release reactive oxygen, the culprit of oxidative stress [51]. IH and resultant oxidative stress have been proposed as a pathogenetic pathway between OSA and disturbance of glucose homeostasis [51], insulin resistance [52], hypercholesterolemia [53], and hyperlipidemia [54]. Similarly, inflammatory cytokines (e.g., tumor necrosis factor-α and interleukin-6) that are triggered by IH and sleep fragmentation have been postulated as a putative mechanism of MS. Inflammatory cytokines may also impair insulin action in peripheral tissues and increase insulin resistance, dyslipidemia, and hypertension in OSA [55]. In conclusion, OSA is linked with metabolic dysregulation in the complex human biological system. IH and sleep fragmentation are suggested to trigger an array of downstream effects (i.e., sympathetic activation, neurohumeral changes, inflammation, and oxidative stress), which are pathophysiological cascades that are common to the pathogenesis of cardiometabolic diseases [16]. However, a specific weakness of our study must be mentioned with respect to pathophysiological aspects, which were not the main focus of the review.

The shared relationship of OSA and MS with obesity should be addressed. Obesity is a major risk factor for OSA because it directly or indirectly contributes to upper airway narrowing during sleep. An example of this situation is by promoting enlargement of soft tissue structures within and surrounding the airway, increasing abdominal fat mass, and recumbent posture [56]. Additionally, obesity, in particular abdominal obesity, is thought to be connected with MS [12]. Our previous study reported that the prevalence of MS in participants with obesity was significantly higher than that in the general population [57]. Therefore, obesity may play a major potential confounding role and should be adjusted in the relationship between OSA and MS. Our meta-analysis showed that obesity, as well as age and sex, could be adjusted as confounding factors in only 12 of 20 included studies. However, we found that all of the studies evaluated obesity only by measuring and calculating BMI, and other accurate imaging examinations (e.g., computed tomography, magnetic resonance imaging) were not performed. We also found that only four studies further adjusted for smoking and alcohol drinking [17, 33–35]. More importantly, none of the studies adjusted for other important risk factors of MS (e.g., educational level, family or personal income, and family history of diabetes or hypertension) [58], which would be expected to reduce the positive association between OSA and MS towards the null value.

We observed a substantial difference among the included studies. An example of this difference was that the normal limit of AHI for diagnosing OSA varied between studies. Most studies used a threshold of over five events/h, and some used 10 events/h [28, 34, 41] or 15 events/h [17, 32, 36]. Furthermore, because MS is a new concept with debatable criteria over a decade [11], two definitions with individual waist circumference criteria to define abdominal obesity in different ethnics were adopted in our meta-analysis. Additionally, adjusted ORs were preferred in the meta-analysis. However, because of the fact that the relationship between OSA and MS was not the primary outcome in some studies, the adjusted ORs were not provided. Therefore, we could only use raw data to calculate the crude ORs in some included studies. In addition, although Papanas et al. [18] suggested that a strong association became non-significant when confounders were considered, the adjusted ORs were not provided and the crude ORs, which exhibited a significant difference, were thus adopted. However, the results of the subgroup analyses confirmed the robustness of association between OSA and MS.

Our study has several potential limitations. First, the included studies were cross-sectional or case–control studies, and none were cohort studies, which could not prove a cause-effect relationship between OSA and MS. However, a beneficial effect of continuous positive airway pressure (CPAP) therapy on MS in patients with OSA could provide more information about the causal relationship [59]. Future randomized controlled trials are required to investigate causality. Second, although the adjusted ORs from each included study were preferred in the meta-analysis, we could not exclude the possibility that our results may have been influenced because adjustment for potential confounders differed in each study. Third, selective outcome reporting remains a possibility. Some studies were speculated to be related to the topic of OSA and MS, but adjusted ORs or raw data were not obtained, despite attempting to contact authors for additional published or unpublished data. Fourth, the two methods that we adopted for assessing the severity of OSA might not be appropriate because they were not formally tested. This could be the possible reason for the large heterogeneity in the meta-analysis of severity of OSA on the risk of MS. Finally, the search was limited to English-language studies only, which had the potential of not including studies in other languages.

## Conclusions

In summary, this meta-analysis of cross-sectional and case–control studies confirms a positive association between OSA and MS, although causality between these two factors has not been demonstrated yet. OSA and the MS are important cardiovascular risk factors and they may act synergistically. With the rapidly growing health problem of OSA and MS, further population- or community-based cohort and randomized controlled studies with adequate adjustment for multiple major confounding factors are required.

## Additional files

Additional file 1:
**Search strategy.** (DOCX 13 kb) Additional file 2: Figure S1. Meta-analysis for all studies including two conference reports that were not subsequently published. Two conference reports referred by Velazquez, 2011 and Papatsimpas, 2011. OR: odds ratio; CI: confidence interval. (TIFF 156 kb) Additional file 3: Figure S2. Funnel plots among all studies including two conference reports that were not subsequently published. Two conference reports referred by Velazquez, 2011 and Papatsimpas, 2011. (TIFF 45 kb) Additional file 4: Figure S3. Galbraith plot. (TIFF 44 kb)

## Abbreviations

OSA: Obstructive sleep apnea
MS: Metabolic syndrome
OR: Odds ratio
CI: Confidence interval
BMI: Body mass index
NOS: Newcastle-Ottawa Scale
AHI: Apnea–hypopnea index
PSG: Polysomnography
NCEP ATP: National Cholesterol Education Program Adult Treatment Panel
IDF: International Diabetes Federation
IH: Intermittent hypoxia
CPAP: Continuous positive airway pressure
CVD: Cardiovascular disease
