# Supplementary material for: The association between obstructive sleep apnea and metabolic syndrome: a systematic review and meta-analysis
Source: BMC Pulm Med. 2015 Sep 21;15:105. doi: 10.1186/s12890-015-0102-3 (PMC4578823; doi:10.1186/s12890-015-0102-3)
Supplement: Additional file 1: — Search strategy. (DOCX 13 kb) [file 12890_2015_102_MOESM1_ESM.docx]

**Additional file 1: Search strategy**

**Example: Pubmed**

01. “obstructive sleep apnea”

02. “obstructive sleep apnoea”

03. “sleep apnea syndrome”

04. “sleep apnoea syndrome”

05. “sleep-disordered breathing”

06. OSA

07. SAS

08. SDB

09. 01 or 02 or 03 or 04 or 05 or 06 or 07 or 08

10. “metabolic syndrome”

11. “insulin resistance syndrome”

12. “metabolic syndrome x”

13. “syndrome x”

14. 10 or 11 or 12 or 13

15. 09 and 14
