# Supplementary figures and images for: The association between obstructive sleep apnea and metabolic syndrome: a systematic review and meta-analysis
Source: BMC Pulm Med. 2015 Sep 21;15:105. doi: 10.1186/s12890-015-0102-3 (PMC4578823; doi:10.1186/s12890-015-0102-3)

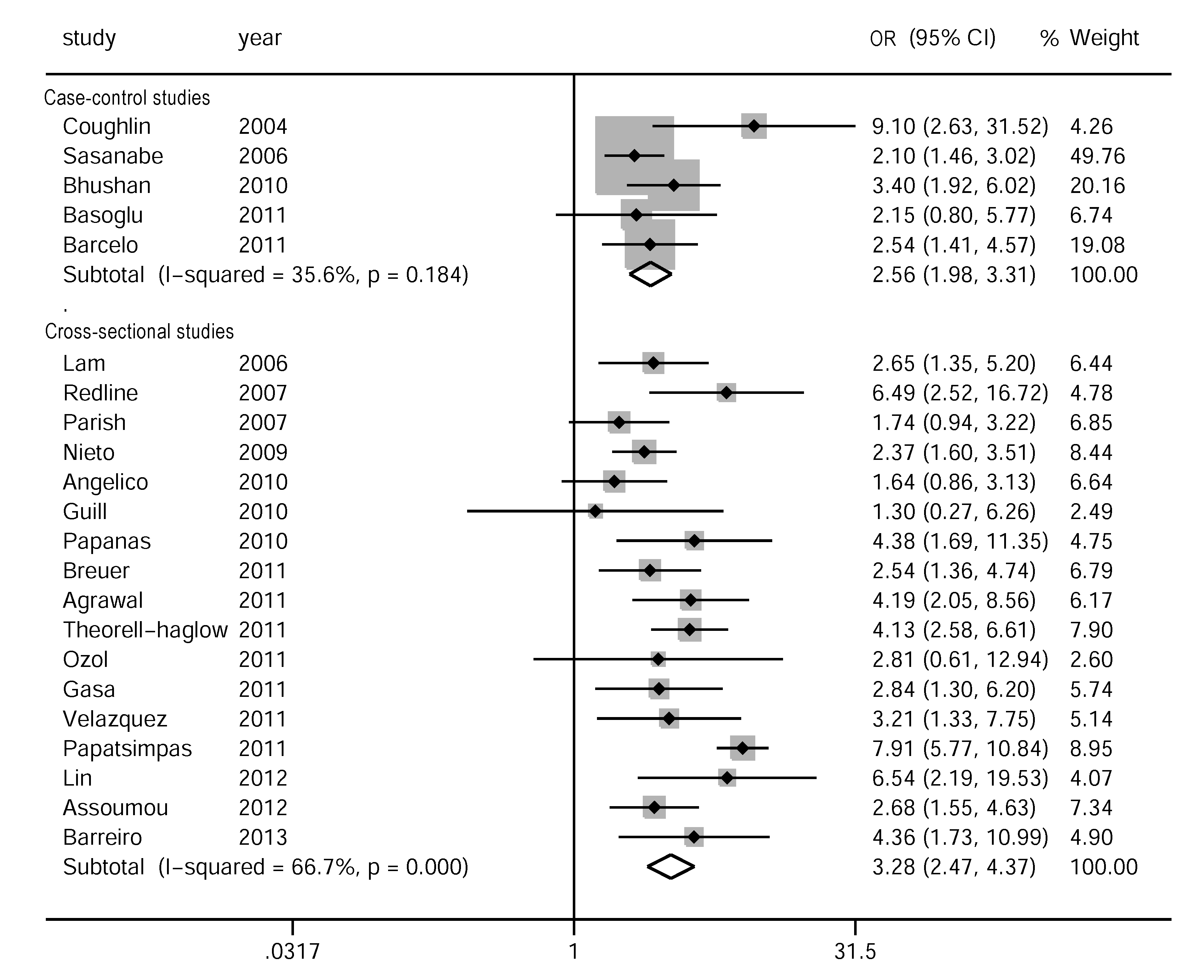

Supplement: Additional file 2: Figure S1. — Meta-analysis for all studies including two conference reports that were not subsequently published. Two conference reports referred by Velazquez, 2011 and Papatsimpas, 2011. OR: odds ratio; CI: confidence interval. (TIFF 156 kb) [file 12890_2015_102_MOESM2_ESM.tiff]

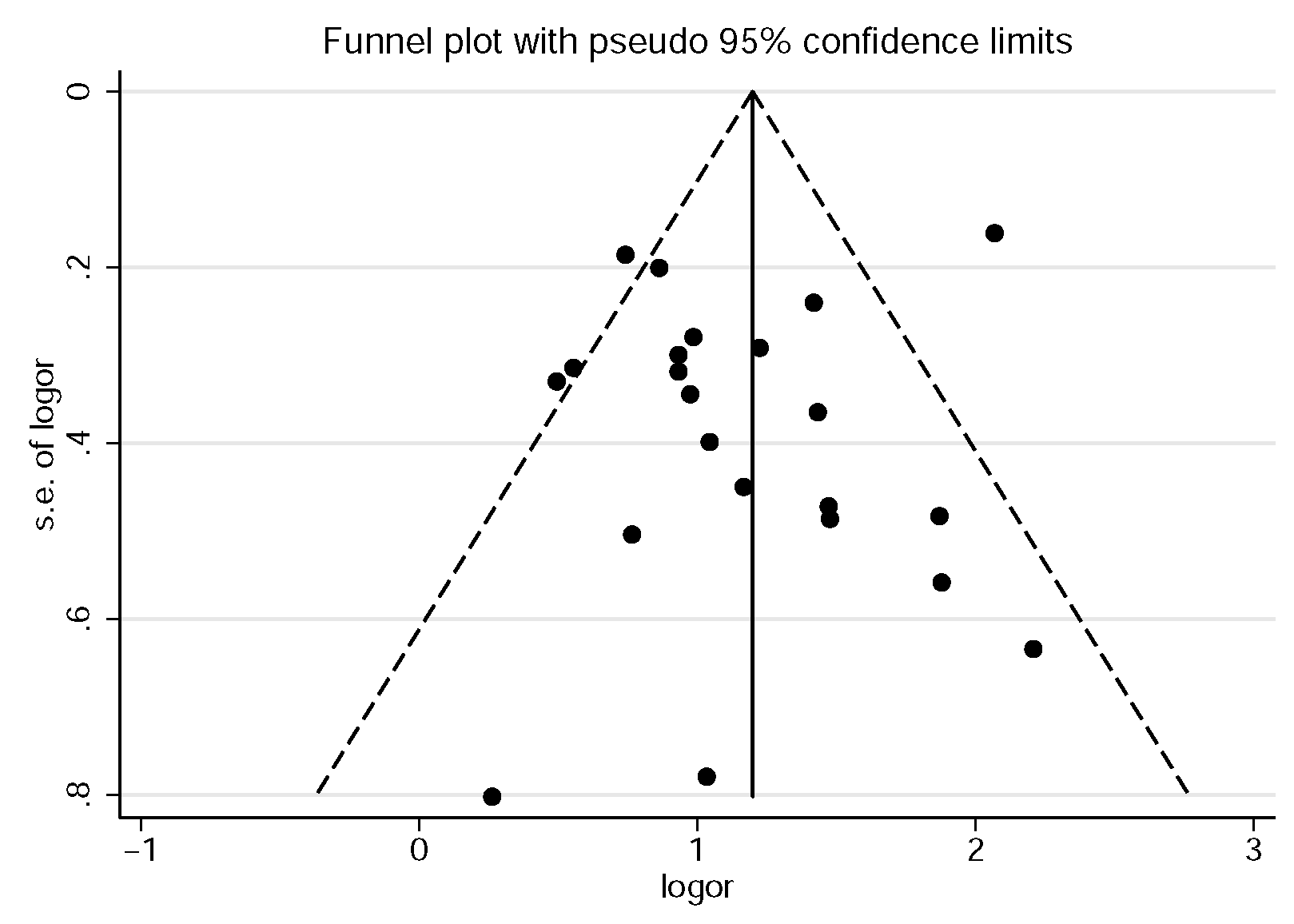

Supplement: Additional file 3: Figure S2. — Funnel plots among all studies including two conference reports that were not subsequently published. Two conference reports referred by Velazquez, 2011 and Papatsimpas, 2011. (TIFF 45 kb) [file 12890_2015_102_MOESM3_ESM.tiff]

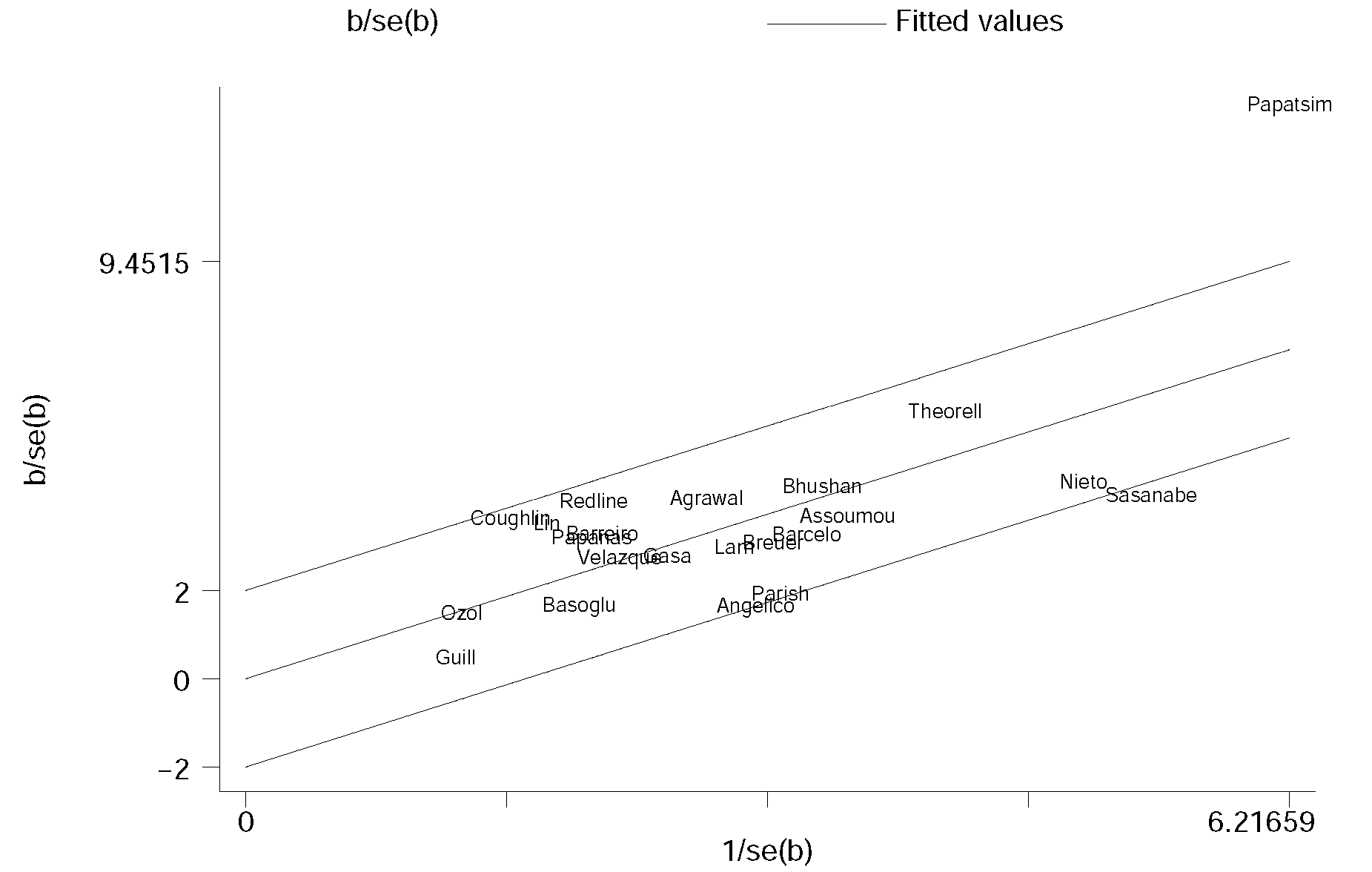

Supplement: Additional file 4: Figure S3. — Galbraith plot. (TIFF 44 kb) [file 12890_2015_102_MOESM4_ESM.tiff]
